# Therapeutic Options for the Treatment of Interstitial Lung Disease Related to Connective Tissue Diseases. A Narrative Review

**DOI:** 10.3390/jcm9020407

**Published:** 2020-02-03

**Authors:** Caterina Vacchi, Marco Sebastiani, Giulia Cassone, Stefania Cerri, Giovanni Della Casa, Carlo Salvarani, Andreina Manfredi

**Affiliations:** 1 PhD Program in Clinical and Experimental Medicine, University of Modena and Reggio Emilia, 41121 Modena, Italy; 2 Rheumatology Unit, University of Modena and Reggio Emilia, Azienda Ospedaliero-Universitaria Policlinico di Modena, 41121 Modena, Italy; 3 Respiratory Disease Unit, University of Modena and Reggio Emilia, Azienda Ospedaliero-Universitaria Policlinico di Modena, 41121 Modena, Italy; 4 Radiology Unit, University of Modena and Reggio Emilia, Azienda Ospedaliero-Universitaria Policlinico di Modena, 41121 Modena, Italy

**Keywords:** interstitial lung disease, connective tissue disease, treatment, clinical trials, immunoppressants, antifibrotic drugs

## Abstract

Interstitial lung disease (ILD) is one of the most serious pulmonary complications of connective tissue diseases (CTDs) and it is characterized by a deep impact on morbidity and mortality. Due to the poor knowledge of CTD-ILD’s natural history and due to the difficulties related to design of randomized control trials, there is a lack of prospective data about the prevalence, follow-up, and therapeutic efficacy. For these reasons, the choice of therapy for CTD-ILD is currently very challenging and still largely based on experts’ opinion. Treatment is often based on steroids and conventional immunosuppressive drugs, but the recent publication of the encouraging results of the INBUILD trial has highlighted a possible effective and safe use of antifibrotic drugs as a new therapeutic option for these subjects. Aim of this review is to summarize the available data and recent advances about therapeutic strategies for ILD in the context of various CTD, such as systemic sclerosis, idiopathic inflammatory myopathy and Sjogren syndrome, systemic lupus erythematosus, mixed connective tissue disease and undifferentiated connective tissue disease, and interstitial pneumonia with autoimmune features, focusing also on ongoing clinical trials.

## 1. Introduction

Interstitial lung disease (ILD) is one of the most serious pulmonary complications of connective tissue diseases (CTDs), with variable clinical impact and severity, often resulting in significant morbidity and mortality. The prevalence reported for connective tissue disease-associated interstitial lung disease (CTD-ILD) is variable according to the different classification modalities among case series and the specific diagnosis [1], with a higher frequency in systemic sclerosis (SSc) and idiopathic inflammatory myopathies (IIM) [2,3]. Recently, the definition of interstitial pneumonia with autoimmune features (IPAF) [4,5] has been proposed for a subgroup of patients with ILD and clinical and/or serological findings suggestive of but not diagnostic of a definite CTD, but this remains to be better investigated. Moreover, ILD may be the first manifestation of CTD, preceding by many years a diagnosis of CTD, representing possibly an incomplete form of CTD [2,6,7].

ILD can be characterized by various patterns of fibrosis on a high-resolution CT (HRCT) scan and in lung biopsy specimens [6]. Common histologic patterns include nonspecific interstitial pneumonia (NSIP), usual interstitial pneumonia (UIP), lymphocytic interstitial pneumonia (LIP) and organizing pneumonia (OP), acute interstitial pneumonia, or unclassifiable pneumonia [1,2,8].

Due to the lack of randomized controlled trials (RCTs) and recommendations, the choice of therapy for CTD-ILD is currently very challenging.

The aim of this review is to summarize the available data and recent advances about therapeutic strategies for ILD in the context of SSc, IIM and Sjogren syndrome (SS), systemic lupus erythematosus (SLE), mixed connective tissue disease (MCTD) and undifferentiated connective tissue disease (UCTD), and IPAF, focusing also on ongoing clinical trials (Table 1).

## 2. Systemic Sclerosis (SSc)

SSc is a CTD characterized by degenerative microvascular phenomena and immune system activation, leading to fibrosis of the skin and internal organs.

ILD is very frequent in patients affected by SSc, reaching 70–90% prevalence, representing the leading SSc-related cause of death [33]. The most common ILD pattern in SSc patients is NSIP, although UIP can also be seen in 25–40% of cases [1,2].

There is no consensus or evidence-based guidelines about the correct timing for the starting of SSc-ILD treatment. Current approaches to SSc-ILD treatment include routine follow-up alone in patients with low or no disease progression. On the contrary, active immunosuppression is required in subjects with progressive ILD [33,34]. Indeed, since initial pulmonary function indexes and the extent of fibrosis on HRCT scans seem to be important determinants of outcome, some staging systems have been proposed to guide the management of non-advanced SSC-ILD based on the prognostic evaluation. Goh et al. have proposed a simple staging whereby extensive disease (>20% HRCT involvement) should require immunosuppressive treatment while limited disease would not should not. When the extent of fibrosis is indeterminate, the forced vital capacity (FVC) should drive the decision to treat, when FVC is <70% predicted, or not to treat. Additionally, the GAP model (Gender, age, and lung physiology model), elaborated in the context of IPF, performed well in a cohort of ILD-SSc patients in predicting mortality [35,36].

Generally, CYC, mofetil mycophenolate (MMF) or rituximab (RTX) are administrated as induction treatment. Then, a maintenance regimen therapy is usually proposed, although no studies have evaluated better maintenance regimens and which patients could benefit from them [37].

### 2.1. Glucocorticoids (GC)

The efficacy of GC therapy is controversial, but they are still empirically used, including in most clinical trials, in association with immunosuppressive drugs. In an observational study by EUSTAR, including 3778 SSc-ILD patients, GCs were used in more than half of patients and even at dosages > 20 mg/day, although the effect of GCs on lung function was only slightly positive in patients with forced vital capacity (FVC) > 75% [34]. Furthermore, an increased risk of scleroderma renal crisis has been described in patients treated with long-term GC therapy of over 10 mg of equivalent of prednisone daily [38].

### 2.2. Cyclophosphamide (CYC)

Two high-quality RCTs have investigated the use of CYC, the Scleroderma Lung Study I (SLS I) and the Fibrosing Alveolitis in Scleroderma (FAST) trial (Table 1). Despite its known toxicity, CYC should be considered for the treatment of SSc-related ILD (SSc-ILD), particularly in patients with progressive ILD [9,11,12,39]. The CYC dose and duration of treatment have to be tailored individually, depending on the clinical condition and response [39].

In SLS I, which included 145 subjects with SSc-ILD, one-year oral CYC treatment was associated with significant but modest improvement in FVC, dyspnea, quality of life, and radiologic aspects [9]. The treatment was limited to one year because of the appearance of adverse events such as leukopenia and hematuria, which were quite common in treated patients. However, the effect of CYC was transient and reversed after 24 months of follow-up [10]. These data were not confirmed in SLS II, which included 142 SSc-ILD patients and compared CYC and mofetil mycophenolate (MMF). In SLS II, the effect of the treatment, both CYC and MMF, was greater in patients with more severe lung or skin involvement [13,40,41]. However, the very limited decline in FVC observed in the placebo cohort suggested a relatively nonprogressive ILD in the population enrolled in the trial.

In another randomized, double-blind, placebo-controlled trial, the FAST trial, Hoyles et al. randomized 45 subjects affected by SSc-ILD. Patients belonging to the treated group received steroids and intravenous CYC for six months, followed by daily oral azathioprine (AZA) [12]. The improvement of FVC at 12 months was modest and did not reach statistical significance because of the small sample size, since only 68% of patients in active treatment and 57% of those who received the placebo completed the trial. Unlike SLS I and II trials, few adverse events were found among the treated patients, without any statistical difference compared to the placebo group, suggesting an improved safety profile of intravenous CYC compared with oral CYC [12].

Considering the results of both RCTs and the fact that the benefit of CYC was mainly due to the inhibition of progression of SSc-ILD, the experts recommended, through the latest European League Against Rheumatism (EULAR) guidelines, that CYC therapy should be mainly considered in patients with progressive lung disease. Furthermore, the tailoring of the CYC dose and the treatment duration should be determined individually, according to the clinical condition and response [39]. Typical protocols suggest CYC treatment for 6–12 months, followed by a switch to another agent for maintenance therapy, usually MMF or AZA [37].

Because of the limited magnitude and duration of CYC benefit, the high rates of adverse effects, and the long-term oncologic risk [42], there is a need for a safer and more effective therapeutic alternative.

### 2.3. Mycophenolate Mofetil (MMF)

Many observational (prospective or retrospective) studies provide encouraging results for the use of MMF in SSc-ILD treatment and led to many experts using this as a first line therapy [43,44,45,46,47,48]. SLS II is the only available randomized trial evaluating the safety and efficacy of MMF in SSc-ILD patients, compared to oral CYC (Table 1) [13]. The authors compared the efficacy and safety of two-year MMF treatment vs. one-year oral CYC followed by placebo for an additional year. MMF treatment led to a significant improvement in FVC and dyspnea in the course of 24 months of follow-up, without a difference between the two treatment groups. The average FVC presented a modest decline between 21 and 24 months in both groups and a complete loss of efficacy was not observed in the CYC group, as occurred in SLS I. Notably, diffusing capacity of the lung for carbon monoxide (DLCO) decreased in both treatment groups, although this finding was significantly greater in the CYC versus the MMF group. The study also demonstrated a greater safety and tolerability of MMF compared with CYC, although no differences in the incidence of infection, bleeding, or death were recorded [13].

Interestingly, EULAR international guidelines did not consider the use of MMF in the treatment of SSc-ILD because they were published before the publication of the results of SLS II study [39].

Two important limitations characterized SLS II: the choice of oral CYC, less tolerated than intravenous CYC [12], and the lack of a placebo group, making comparisons between the MMF effect and the natural history of SSc-ILD difficult. To overcome this limitation, Volkmann et al. compared the outcomes for patients assigned to the MMF group of SLS II with patients assigned to the placebo group of SLS I [14]. Taking into account the limits inherent in the design of the study, MMF showed long-lasting efficacy on lung function’s parameters and dyspnea and a high safety profile.

### 2.4. Azathioprine (AZA)

The efficacy of AZA was investigated only in the FAST trial (Table 1), where it was administered as maintenance therapy after intravenous CYC [12] and in other unblinded studies [15,49,50]. Despite this, in clinical practice AZA is considered a well-tolerated, alternative agent for maintenance therapy in SSc-ILD, after CYC or MMF therapy [51,52].

### 2.5. Rituximab (RTX)

Several studies have suggested a possible role of RTX in the management of SSc-ILD [16,53,54,55,56,57,58,59], including a randomized controlled study (Table 1) [16,17] and a nested case-control study [54]. Available data showed encouraging short- and long-term results, with an acceptable safety profile.

The first open-label, randomized, controlled study to evaluate the efficacy of RTX in patients with SSc-ILD was performed by Daoussis et al. Fourteen patients were enrolled, eight of them treated with four weekly pulses of RTX (375 mg/m^2^) at the baseline and after six months. The remaining control group continued their previously administered treatment (GC, CYC, MMF, or bosentan). They reported an improvement in lung function and radiological stabilization after one year of follow-up in treated patients compared to the control group [16]. Subsequently, they described the long-term efficacy, with an acceptable safety profile of RTX in SSc-ILD, at a seven-year follow-up [55]. Interestingly, all patients deteriorated after RTX cessation.

Jordan et al. conducted the first multicenter nested case control observational study, including 63 patients, of whom nine presenting with ILD. Treatment with RTX improved skin fibrosis and stabilized lung function parameters in patients presenting ILD, showing an acceptable safety profile even from the infectious point of view [54].

The RECOVER trial (NCT01748084), a randomized triple-masking study, has recently been terminated. The primary purpose was to determine whether RTX is effective in the treatment of articular symptoms related to SSc polyarthritis. Secondary outcomes included the assessment of RTX’s efficacy on lung function, but the results are not yet available (Table 1) [17].

RECITAL trial (NCT01862926) is an ongoing double-blind RCT comparing intravenous RTX with intravenous CYC in patients affected by various CTDs, including SSc, with the change in FVC at 24 weeks as primary outcome [31].

### 2.6. Hematopoietic Stem Cell Transplantation (HSCT)

Hematopoietic stem cell transplantation is a potentially efficacious cell-based treatment even if it is associated with high mortality, mainly due to infections [18,19,20,37,39,60]. For this reason, it should be considered in specialized centers for the treatment of selected patients with rapidly progressive SSc at risk of organ failure [39]. Autologous CD34^+^ stem cells are re-infused to rescue an autoreactive immune system previously ablated with high-dose CYC and anti-thymocite globulin, with or without total body irradiation. The major studies are the Autologous Stem Cell Transplantation (ASTIS) trial (Table 1) [18], the Autologous Stem Cell Systemic Sclerosis Immune Suppression (ASSIST) trial (Table 1) [19], and the Scleroderma: Cyclophosphamide or Transplantation (SCOT) trial (Table 1) [20]. This last trial tested a different therapeutic approach, consisting of myeloablation with total-body irradiation followed by reconstitution with a CD34+ selected autograft versus CYC [20].

The first single-center trial, the ASSIST trial, including 19 patients with SSc and SSc-ILD, showed that HSCT was superior to CYC therapy with respect to an improvement in skin score and lung volume [19]. No significant effect on the diffusing capacity of the lungs for carbon monoxide could be demonstrated. The ASTIS trial compared HSCT with CYC pulse therapy in 156 patients with early diffuse SSc. HSCT was associated with increased treatment-related mortality in the first year, but significantly improved long-term event-free survival and overall survival. HSCT therapy resulted in significant improvement in the FVC and total lung capacity (TLC) at a two-year follow-up. Regarding safety, it is noteworthy that eight deaths (10.1%) in the HSCT arm occurred. Causes of treatment-related deaths included EBV, lymphoma, heart failure, myocardial infarction, and acute respiratory distress syndrome.

No significant effect on DLCO could be found in the ASSIST or ASTIS trial. In the SCOT trial myeloablative autologous hematopoietic stem-cell transplantation, patients achieved long-term benefits in patients with SSc, including improved event-free and overall survival, at a cost of increased toxicity. The primary end-point was a global rank composite score comparing participants with each other on the basis of a hierarchy of disease features assessed at 54 months, including FVC. Data regarding pulmonary function alone were not available [20].

### 2.7. Lung Transplantation

Currently, the data regarding survival after lung transplantation are poor and contradictory [61,62,63,64,65,66,67,68]. In two retrospective analyses, survival rates appeared to be similar to those observed in subjects with other fibrotic ILD [64,69]. Possible negative prognostic factors were identified: renal impairment, possible onset of skin ulcerations and consequent complicated surgical wound repair, arrhythmias, and older age [65,66]. Furthermore, esophageal involvement (reflux and dysmotility) is a controversial risk factor for complications and poor survival in these patients [33,63,65,70].

### 2.8. Tyrosine Kinase Inhibitor

Based on preclinical evidence and clinical similarities between idiopathic pulmonary fibrosis (IPF) and SSc-ILD, a phase III double-blinded, randomized, placebo-controlled study (SENSCIS trial) has been performed with the purpose of evaluating the efficacy and safety of Nintedanib for at least 52 weeks in patients presenting SSc-ILD (Table 1) [21]. Nintedanib targets receptors involved in the pathogenesis of SSc-ILD and impacts the fundamental processes of lung fibrosis in relevant animal models. In preclinical studies, the drug was demonstrated to reduce the tissue density, fibrosis score, and myofibroblast count. Moreover, in an animal model of SSc, Nintedanib normalized the distorted pulmonary vascular architecture and reduced the vessel wall thickness and number of occluded vessels [71].

However, in the SENSCIS trial, Nintedanib significantly reduced the annual rate of decline in FVC assessed over 52 weeks (−52.4 versus −93.3 mL/year; *p* = 0.04), even if the relative reduction in FVC decline was similar in the two groups (44% and 49% for Nintedanib and the placebo, respectively) [21,72]. Moreover, the *p* values yielded ranged from 0.06 to 0.10 after correction with multiple-imputation sensitivity analyses for missing data [21,72].

The SENSCIS trial had some major limitations, however: it included a broad range of patients with SSc-ILD, including almost 50% of patients with limited cutaneous SSc and almost 50% taking MMF at the baseline.

The annual rates of change in FVC among the patients who were receiving MMF at baseline were −40.2 mL per year in the nintedanib group and −66.5 mL per year in the placebo group, and the corresponding rates among the patients who were not receiving mycophenolate at baseline were −63.9 mL per year and −119.3 mL per year.

The decline in FVC in the placebo group, as well as the magnitude of the effect of nintedanib, is influenced by concurrent immunosuppressive treatment with MMF, suggesting the effectiveness of MMF on lung involvement in SSc. Nevertheless, since patients have not been stratified for ILD progression or ILD histological/radiological pattern, the mechanism of MMF and the population who could potentially benefit by the drug can be only supposed [21].

Moreover, Nintedanib was observed to have no effect on skin fibrosis, or on health-related quality of life. The drug was approved by the FDA for the treatment of SSc-related ILD, but further studies are needed to better characterize the profile of SSc patients to be treated with Nintedanib [21].

Imatinib did not demonstrate efficacy in SSc-ILD and in general was not well tolerated [22,23]. In open-label clinical trials, Nilotinib and Dasatinib treatment did not show significant clinical efficacy [24].

### 2.9. Pirfenidone

Pirfenidone is an antifibrotic agent approved for the treatment of IPF. After the publication of some weak and initial evidence [73,74], the combination therapy pirfenidone‒MMF showed an acceptable tolerability profile in SSc-ILD in an open-label, 16-week trial (Table 1) [25]. Scleroderma lung study III is ongoing to investigate the effects of pirfenidone vs. placebo in SSc-ILD patients who are receiving MMF (Table 1) [26].

### 2.10. IL-6 Blockade

The Phase II faSScinate trial showed no significant difference between tocilizumab and placebo in the primary endpoint of change in modified Rodnan skin score, but exploratory analyses suggested that tocilizumab may be associated with a clinically relevant improvement in lung function (Table 1) [27]. In the Phase III focuSSced trial, in exploratory analyses, treated patients showed a minor decline in FVC; in particular, the mean change from the baseline in FVC at week 48 was −0.4% predicted in the tocilizumab group versus the −4.6% predicted in the placebo group, and the proportion of patients with a decline in FVC of >10% predicted at week 48 was 5.4% with tocilizumab and 16.5% with the placebo (Table 1) [28].

## 3. Idiopathic Inflammatory Myopathies (IIM)

IIM, namely polymyositis (PM), dermatomyositis (DM), antisynthetase syndrome (ASSD), and clinically amyopathic dermatomyositis, are a heterogeneous group of CTDs mainly characterized by skeletal muscle inflammation but also by multiple organ involvement, among which ILD represents the most common non-musculoskeletal manifestation of the disease [2,75,76,77,78,79,80]. The most frequent ILD pattern in IIM patients is NSIP, but co-existing patterns of organizing pneumonia (OP) and diffuse alveolar damage (DAD) have been reported; UIP has been described with a lower prevalence among these subjects [77]. Especially in presence of UIP pattern, differential diagnosis with IPF can be challenging if myositis is clinically absent, as in amyopathic dermatomyositis. In these cases, an accurate serological assessment is mandatory.

No evidence-based guidelines exist regarding IIM-ILD therapy regimens, including dosage recommendations. Since clinical characteristics, treatment response, and prognosis are highly variable among patients, it is critical to predict future outcomes in DM patients before the initiation of management. PM-ILD seems to respond to conventional treatment better than DM-ILD [80,81]. Regarding this latter, anti-MDA5 antibody is a myositis-specific autoantibody related to this form and it is associated with GC resistance and poor prognosis. On the contrary, anti-aminoacyl tRNA synthetase antibodies, a group of myositis-specific autoantibody related to ASSD, lead to greater GC treatment response even if they are associated to a higher risk of disease relapsing [82,83,84].

Available data from observational studies suggest that the initial therapeutic choice should depend on the ILD severity and modality of presentation; there is no strong evidence regarding the superiority of a particular agent to others. A severe and rapidly progressive ILD requires a more aggressive therapy such as a high dose of GC, CYC, calcineurin inhibitors, or RTX in case of refractoriness to conventional therapies. Otherwise, patients with mild disease or a chronic presentation can be treated with a combination of GCs and MMF or AZA [77,78].

### 3.1. Polymyositis (PM), Dermatomyositis (DM), Amyopathic Dermatomyositis

#### 3.1.1. Glucocorticoids (GC)

Despite poor evidences, GC are still the first-choice treatment for IIM-ILD patients [2,85]; immunosuppressants are frequently added in nonresponding patients and to reduce the GC-related side effects [77,86].

#### 3.1.2. Cyclophosphamide (CYC)

A systematic review demonstrated that oral or intravenous CYC significantly improves pulmonary function tests and HRCT scores in a high percentage of patients [76,87,88,89,90,91] after six or 12 months of therapy, also associated with an improvement in survival rate [87]. On the contrary, Meyer et al. found no significant improvement in pulmonary function tests and HRCT scores after CYC treatment, reporting that the CYC effect seems to be higher if started in the early stages of the disease [88]. No sub-analyses have been performed to determine the efficacy of CYC in patients with ASSD (107/193 subjects).

#### 3.1.3. Mycophenolate Mofetil (MMF) and Azathioprine (AZA)

Data supporting the effectiveness of AZA and MMF, in particular as a maintenance therapy after induction or for patients with mild ILD, are limited to small case series and case reports [90,91,92,93,94,95,96]. However, Mira-Avendano reported the same efficacy for oral CYC, AZA, and MMF in PM/DM-ILD and ASS-ILD, concluding that all three drugs were useful for stabilizing lung function and allowing GC dose tapering [90]. Furthermore, Huapaya et al. recently conducted a retrospective study on 66 patients with IIM-ILD treated with AZA and 44 with MMF describing a significant improvement of FVC (%) with both drugs, and also of DLCO (%) within subjects treated with AZA. After 36 months, patients treated with AZA received a lower prednisone dose than those treated with MMF [97].

#### 3.1.4. Calcineurin Inhibitors

Many retrospective studies, regarding both cyclosporine and tacrolimus, suggested their efficacy in treating IIM-ILD [94,98,99,100,101,102]. In a retrospective study, Shimojima et al. observed that patients treated in association with GC in the early phase of the disease and with an adequate blood concentration showed a better response [102].

#### 3.1.5. Methotrexate (MTX)

MTX has been used for the treatment of IIM-ILD despite the absence of pulmonary-specific evidence. In fact, MTX presents a good safety profile, although its association with idiosyncratic drug-related hypersensitivity pneumonitis is well known [103].

### 3.2. Antisynthetase Syndrome (ASSD)

ILD prevalence in ASSD is high, reaching 70–100% in some case series, and can precede muscle symptoms or occur without clinical myositis in about 10–20% of patients [104]. PL-7 and PL-12 autoantibodies are often associated with more severe ILD.

ASSD-ILD shows greater treatment responsiveness and risk of recurrence in comparison with other IIM-ILD [2,105].

Mira-Avendano et al. retrospectively evaluated the efficacy of CYC, AZA, and MMF in ILD with approximately 50% of the patients positive for Jo-1, and found no clear differences between the drugs [90].

In an Italian study, 17 Jo-1-positive patients with ILD received cyclosporine after the failure of GC monotherapy. After one year of follow-up, their DLCO, FVC, and HRCT scores all improved. In a systematic review pooling data from four retrospective studies that evaluated the effect of tacrolimus in Jo-1-positive, IIM-associated ILD, the authors reported that over 80% of subjects showed improvement or stabilization of FVC and DLCO [87,106,107,108,109,110,111,112,113,114].

Regarding CYC, there are some retrospective data regarding few patients. In the study by Ingegnoli et al., 15 patients with Jo-1-positive ILD, seven received steroids + cyclosporine and eight received steroids and intravenous cyclophosphamide. HRCT worsened in half of the CYC group and in 5/7 (71%) patients receiving cyclosporine [115]. On the other hand, in the study by Chen et al., 3/5 subjects responded positively to this therapy [116].

Recently, Hozumi et al. demonstrated, by mean of propensity score analysis, that first-line combination of GC +calcineurin inhibitors treatment for patients with ASSD significantly improved the progression-free survival compared with GC monotherapy, although there was no significant difference regarding long-term survival [117].

Favorable responses to RTX have been reported in case reports and retrospective uncontrolled studies, in particular ASSD-ILD [118,119,120,121,122,123,124].

Keir et al. described four anti-Jo-1 patients with a good response to RTX in terms of respiratory function [122]. In another series, 7/11 patients presenting with ASSD experienced a stabilization (in one case) or improvement (in six cases) of respiratory function six months after receiving RTX [123]. The same authors reported long-term data on 24 patients with >12 months of follow-up after RTX [125]. In this study, the median percentages of FVC and DLCO increased by 24% and 17%, respectively. One of the limitations of this study was that RTX was never administered as monotherapy; in particular, 10 of the 12 patients with acute disease also received CYC. Furthermore, different kinds of maintenance therapy were started 0–3 months after the first RTX cycle. Allenbach et al. conducted the first open-label phase II trial evaluating RTX in 10 refractory anti-Jo-1 subjects who received RTX at days 0 and 15, and after six months. At month 12, FVC and DLCO were stable in four patients and improved in five (50%) (Table 1) [29].

A randomized, controlled pilot 24 weeks trial, the ATtackMy-ILD trial, is ongoing to investigate the efficacy and safety of subcutaneous Abatacept in treating ASSD-ILD in comparison to placebo. Primary efficacy outcome will be FVC (%) change from baseline and, within secondary outcomes, time to progression free survival, patient reported dyspnea scores and time to improvement in FVC% considering a change of at least 10 points. Investigators are currently recruiting subjects [30].

### 3.3. Others (Intravenous Immunoglobulins, Plasmapheresis, Lung Transplantation) in IIM and ASSD-ILD

Data supporting a role for intravenous immunoglobulins in the management of IIM-ILD are only anecdotal, yet their use has been suggested for rapidly progressive ILD, in refractory forms, or in patients with a contraindication to immunosuppressants [126,127,128].

The role of plasmapheresis is still unclear. Its use has been reported in two cases of ASSD-ILD as an emergency intervention before immunosuppressive therapy became effective [129,130].

Lung transplantation is the last treatment option in end-stage IIM-ILD, as reported in some anecdotal cases [131,132,133,134]. Courtwright et al. reported that adjusted survival was not significantly different among subgroups of IIM-ILD patients compared with IPF patients after lung transplantation [135].

## 4. Primary Sjogren Syndrome (pSS)

pSS is a CTD characterized mainly by lymphocytic infiltration of salivary and lacrimal glands, resulting in xerostomia and xerophthalmia. pSS-related ILD is the most frequent lung involvement and is associated with a reduced quality of life and physical capacity. Furthermore, it is responsible for 42–90% of deaths [7,136]. NSIP is the most frequent ILD pattern in pSS while UIP is only occasionally observed. Noteworthy, LIP, even if rarely detected, is typically associated to pSS [7,136].

Nowadays, the available data are not adequate to establish an evidence-based treatment strategy in pSS-ILD; in particular, it is still not clear what kind of pSS-ILD patients need: immunosuppressive therapy or only observation. For patients presenting with mild or indolent ILD, as seen in some cases of LIP, a wait-and-see strategy could be appropriate. In general, in patients affected by pSS-ILD, the first-line therapy should be GC, alone or in combination with AZA or other immunosuppressants. In refractory cases, RTX should be considered [137,138].

### 4.1. Glucocorticoids (GCs) and Conventional Immunosuppressive Agents

GCs are commonly used as a first-line therapy, with an initial dosage of 0.5–1 mg/kg/d, according to the severity and activity of ILD [136,139]. GCs can be used in monotherapy or in association with other immunosuppressive agents such as CYC, MMF, or AZA in order to minimize steroid-related adverse effects and/or improve the response to GC.

Patients presenting with NSIP, OP, and LIP patterns seem to show a better response to immunosuppressive treatment, in particular to steroid therapy, than subjects presenting with a UIP pattern [136,140].

### 4.2. Rituximab (RTX)

RTX seems to be a safe and useful drug for the treatment of pSS, in particular in patients with systemic involvement [138,141,142], but data regarding pSS-ILD treatment are lacking. Lung disease improvement has been reported only in a small case series [143,144,145].

Gottenberg et al. collected data from the French AutoImmune and RTX Registry, describing eight patients with pSS-ILD treated with RTX. Six patients with a good response to the first RTX course were treated again (with two to nine additional cycles), while the other two patients maintained a response even 30 months after a single RTX cycle [143].

### 4.3. IL-6 Blockade, Tocilizumab

The use of tocilizumab for the treatment of pSS-ILD is anecdotal. Justet et al. reported a case of steroid-resistant OP associated with arthritis in a pSS male patient that improved with Tocilizumab [146].

## 5. Systemic Lupus Erythematosus (SLE)

SLE is a systemic autoimmune disorder involving multiple organs, including the lungs, and is characterized by the production of autoantibodies against nuclear antigens and immunocomplexes. NSIP is the most common ILD pattern, but LIP and OP have also been described in association with SLE, while UIP appears to be very uncommon [2].

The SLE-ILD treatment strategy is based on expert opinion; there are no evidence-based guidelines or clinical trials available. The first-line induction regimen typically includes corticosteroids, alone [147] or in association with other immunosuppressive agents such as CYC or MMF. Maintenance therapy usually includes AZA or MMF, which are also used in the management of mild‒moderate disease [148,149]. RTX has been suggested as a second-line therapy [122,148]. Yang et al. reported the first and only case of a 54-year-old Chinese woman diagnosed with SLE-associated ILD presenting with a dry cough and dyspnea and successfully treated with prednisone and pirfenidone. Furthermore, the treatment led to the improvement of a concomitant malar rash [150].

## 6. Mixed Connective Tissue Disease (MCTD)

MCTD is an overlapping condition characterized by the combined presence of serum Anti-ribonucleoprotein (anti-RNP) antibodies and select clinical features of SSc, SLE, rheumatoid arthritis (RA), and PM/DM.

In MCTD, ILD is usually SSc-like, but progression is slow in the majority of patients. However, as in the other CTDs, ILD is associated with an increased risk of mortality [151].

No controlled data are available regarding MCTD-ILD treatment, but immunosuppressive regimens are required, as in other CTDs [137,152,153]. Conventional therapies for MCTD-ILD include a combination of corticosteroids, which are often efficacious, and steroid-sparing agents, such as CYC and AZA [151,154,155].

## 7. Undifferentiated Connective Tissue Disease (UCTD)

Undifferentiated connective tissue disease (UCTD) is a systemic autoimmune disease characterized by clinical and serological features typical of other CTD, but not fulfilling any of the existing classification criteria [4]. Lung involvement in UCTD is not widely explored in the literature; we report single Centre data showing a high prevalence of inconsistent with (UIP) pattern and a correlation of ILD with Raynaud’s phenomenon, ocular dryness, and antinuclear antibodies [4,156].

Recently, the term IPAF has been introduced to describe a condition characterized primarily by ILD associated with features (clinical, serological, and/or morphological) suggestive of an autoimmune disease. The statement reported research classification criteria not usable in clinical practice with possible detection also of patients classifiable as UCTD [4].

Nevertheless, diagnosis of IPAF represents an inclusion criterium in some ongoing trials for anti-fibrotic in IP other than IPF [32,157].

A multidisciplinary approach, including rheumatologists, pulmonologists, radiologists, and pathologists, is mandatory to correctly classify these patients and set up an appropriate immunosuppressive treatment strategy [4,158].

## 8. Acute Exacerbation (AE)

Acute exacerbation (AE) of ILD is a potentially fatal condition and is defined as an acute, clinically significant respiratory deterioration that develops within less than one month and is characterized by evidence of new widespread alveolar abnormalities detected on HRCT that present histopathologically as diffuse alveolar damage [159]. Furthermore, clinical causes like fluid overload, left heart failure, or pulmonary embolism must be excluded [159]. AE is better described in patients affected by IPF, but is also reported in other forms of ILD, included ILD-CTDs. The estimated one-year incidence of AE-CTD/RA ranges from 1.25% to 9.4% [152,153,154].

### 8.1. Pharmacological Treatment

Although there are no evidence-based guidelines, the management of AE in patients affected by IPF and CTDs typically requires a global approach including pharmacological treatment and supportive care.

The treatment is usually based on high-dose corticosteroids in monotherapy or in association with immunosuppressants, such as cyclophosphamide, oral tacrolimus, or cyclosporine. Furthermore, these patients require broad-spectrum antibiotics [159,160,161,162,163]. Other studies also identified a positive effect of a treatment with RTX with plasma exchange and intravenous immunoglobulin, polymyxin B-immobilized fiber column perfusion in patients diagnosed with IPF and intravenous thrombomodulin in CTD patients [159,164]. Recently, the effective use of Nintedanib in AE in IPF patients has been described in two patients [165,166], and, of note, in INPULSIS-2 trial there was a significant difference between the nintedanib and placebo groups in the time to the first acute exacerbation (hazard ratio, 0.38; 95% CI, 0.19 to 0.77; *p* = 0.005), even if the reliability of this result is still controversial [167,168].

### 8.2. Supportive Care

Supportive care includes in particular palliation of symptoms, mechanical ventilation, and supply of oxygen in hypoxemia. There are still different opinions on the length of this treatment, in particular regarding the use of mechanical ventilation. The decision about this latter has to be made case-by-case together with the physician, the patient, and the family and in accordance with the individual goals of care. As a bridge to lung transplantation, mechanical ventilation or extra-corporal membrane oxygenation may be appropriate and successful in selected patients [159,160].

## 9. Non-Pharmacological Treatments

No studies are available for oxygen supplementation and pulmonary rehabilitation in ILD related to CTDs, but several data are available for IPF.

Pulmonary rehabilitation seems to be safe for patients with ILD. Despite the quality of evidence was low, short-term benefits in functional exercise capacity, dyspnea, and quality of life are usually observed after pulmonary rehabilitation [169]. Exercise interventions can consist of walking, upper and/or lower extremity ergometry, strength training, inspiratory muscle training, and breathing exercises [170].

Oxygen supplementation is usually prescribed when oxygen saturation is <88% at rest, but there are contrasting evidence in IPF about its role in improving dyspnea and exercise capacity [169,170].

## 10. Ongoing Randomized Trials

The RECOVER trial (NCT01748084), a randomized, triple-masking study, is now ongoing, with the primary purpose of determining the efficacy of RTX in the treatment of joint symptoms related to SSc polyarthritis. The possible effect on lung function in patients with ILD is a secondary outcome of the study (Table 1) [17].

RECITAL (NCT01862926) is a United Kingdom-based, multicenter, prospective, randomized, double-blind, and double-dummy controlled trial comparing intravenous RTX (1000 mg), given twice at an interval of two weeks, with monthly intravenously CYC at a dose of 600 mg/m^2^, in patients with ILD due to SSc, IIM (including ASSD), or MCTD. A total of 116 individuals will be randomized 1:1 to each of the two treatment groups, with stratification based on underlying CTD, and will be followed for a total of 48 weeks from the first dose. The primary endpoint for the study will be the change in FVC at 24 weeks. Secondary endpoints include safety, change in FVC at 48 weeks, as well as survival, change in oxygen requirements, total 48-week corticosteroid exposure, and utilization of healthcare resources (Table 1) [31].

SLS III (NCT03221257) is an ongoing phase II, multicenter, double-blind, randomized, and placebo-controlled clinical trial addressing the treatment of patients with active and symptomatic SSc-ILD. Patients who are either treatment-naive or with a recently started treatment will be randomized in a 1:1 assignment to receive for 18 months either oral MMF and a placebo or a combination of oral MMF and oral pirfenidone. The primary hypothesis is that the rapid onset and antifibrotic effects of pirfenidone will complement the delayed anti-inflammatory and immunosuppressive effects of MMF, to produce a significantly more rapid and/or greater improvement in lung function over time than occurs in patients receiving control therapy with MMF and the placebo (Table 1) [26].

Abatacept for the Treatment of Myositis-associated Interstitial Lung Disease (ATtackMy-ILD) (NCT03215927) is a randomized, controlled pilot trial investigating the efficacy and safety of subcutaneous Abatacept in treating ASSD-ILD in comparison to placebo. The primary outcome criteria for efficacy will be the FVC (%) change from the baseline to week 24 while secondary outcomes include time to progression free survival, patient reported dyspnea scores and time to improvement in FVC% considering a change of at least 10 points. This trial is still in recruiting phase. Subjects will be randomized 1:1 to receive placebo for 24 weeks or subcutaneous injection of abatacept 125 mg weekly for 24 weeks. In a 24-week optional follow up phase all subjects receive abatacept 125 mg weekly. Study Completion Date is scheduled for May 2021 [30].

## 11. Conclusions

ILD represents one of the most severe clinical manifestation of CTDs with a deep impact on the prognosis in terms of morbidity and mortality. Nevertheless, with the exception of Ssc, in CTDs validated screening program for early detection of ILD is not available with a significant delay of diagnosis and underestimation of the prevalence and incidence. Furthermore, although CTD-ILD represents an important current field of study for both rheumatologists and pulmonologists, there is still lack of prospective data about prevalence, follow-up, and therapeutic approach.

A direct consequence of this lack of knowledge is that the treatment strategy of CTD-ILD is challenging. With the exception of SSc, the therapeutic approach to CTD-related ILD is usually based on very low-quality studies, retrospective or inconsistent data, and expert opinion (Table 1 and Table 2). Therefore, available data on SSc are generally extended to all CTDs. Heterogeneity of clinical expression of lung involvement in CTDs, variability of onset manifestations, the often-unpredictable disease course (ranging from a slow, progressive decline in lung function over time to a rapidly progressive course or acute exacerbation), and the absence of well-defined outcomes could contribute to the difficulties in planning RCTs [3]. Recently, OMERACT CTD-ILD Working Group elaborated a consensus in order to establish the domains for patient inclusion in RCTs in CTD-ILD and to define clinically meaningful progression [3].

Studies cited in this review, including the RCTs, prospective, nested case control, and retrospective trials, are classified on the basis of the study design in Table 2.

With this background, therapeutic approach in clinical practice is usually based on the use of steroids and immunosuppressive drugs, mainly CYC, MMF, and AZA, both for steroid-sparing purposes and for unresponsive patients. Of interest, many authors included RTX among the possible first-line therapeutic choice in CTD-ILD, even if strong evidence is still not available [16,17,29,31,53,54,55,56,57,58,59,122,125,143,144,145]. Finally, the recent publication of the encouraging results of the INBUILD trial has highlighted a possible effective and safe use of Nintedanib in patients presenting with progressive fibrosing ILDs other than IPF, including subjects with CTD. This trial is a randomized, double-blind and placebo-controlled phase III study that showed a significantly lower annual rate decline in the FVC among patients who received Nintedanib in comparison to subjects who received the placebo [32].

The correct clinical and therapeutic management of this patients should be defined in the context of a multidisciplinary team including rheumatologist, pulmonologist, and thoracic radiologist, taking in account clinical and functional impairment, physiologic or radiographic progression, underlying CTD, comorbidities, patient age, and ability to comply with therapy and monitoring [3,6].

## 12. Highlights

- The correct clinical and therapeutic management of CTDs-ILD needs a multidisciplinary approach including expert rheumatologist, pulmonologist, and thoracic radiologist;
- Treatment with conventional or biologic disease-modifying antirheumatic drug (DMARDs) is often based on expert-opinion and low-quality studies in CTD-ILD;
- Randomized controlled studies are available only for RTX, CYC, MMF, TCZ, and anti-fibrotics, mainly in SSc and sometimes without conclusive results;
- A possible role of antifibrotic treatment in CTDs-ILD has been supposed by some Authors; the INBUILD study demonstrated the efficacy of nintedanib in secondary form of ILD, including CTD;
- Further prospective studies are mandatory to investigate the efficacy and safety of the different treatment regimens in CTDs-ILD;

## Figures and Tables

**Table 1:** Treatment of interstitial lung disease in connective tissue disease.

Systemic Sclerosis								
Trial	Year	Population	Phase	Follow-Up	Drug Investigated		Outcome	Results
SLS I [9]	2006	145 SSc-ILD	Phase III	12 Mo *	CYC	Oral CYC [72 pt] vs. PBO [73 pt]	I: FVC (%); II: TLC (%), PR-D, DLCO (%)	Oral CYC was associated with significant but modest improvement in FVC (%) compared with PBO and was associated with improvements in TLC (%), PR-D but not in DLCO (%).
SLS I, FU extension [10]	2007	145 SSc-ILD	Phase III	24 Mo *	CYC	Oral CYC [72 pt] vs. PBO [73 pt]	I: FVC (%); II: TLC (%), PR-D, DLCO (%)	At a 24 month follow up, except for a sustained impact on dyspnea, the effects on FVC and TLC were no longer apparent.
SLS I ^§^ [11]	2009	98 SSc-ILD	Phase III	12 Mo	CYC	Oral CYC [49 pt] vs. PBO [49 pt]	I: FVC (%), HRCT aspects (GGOs, FIB, Hcs scored on a scale of 0 to 4); II: TLC (%), DLCO (%)	At the end of FU, FIB was significantly worse in the PBO group than in the CYC group (*p* = 0.014) and these differences correlated significantly with FVC, TLC, and PR-D. No differences were noted in terms of GGOs and Hcs.
FAST trial [12]	2006	45 SSc-ILD	N.A.	12 Mo	CYC-AZA	PDN + iv CYC + oral AZA as maintenance therapy [22 pt] or PBO [23pt].	I: FVC (%), DLCO (%); II: HRCT (extent and pattern), PR-D	The improvement in terms of FVC (%) at the end of FU was modest but did not reach the statistical significance. Secondary outcome was not reached.
SLS II [13]	2016	126 SSc-ILD	Phase II	24 Mo	MMF vs. CYC	MMF for 24 months [63 pt] vs. oral CYC for 12 months followed by PBO for 12 months [63 pt]	I: FVC (%); II: DLCO (%), PR-D, quantitative HRCT fibrosis scores.	Both MMF and CYC treatment resulted in significant improvements in FVC (%), DLCO (%), HRCT, PR-D.
SLS I, II ^§^ [14]	2017	122 SSc-ILD	N.A.	24 Mo	MMF	SLS II-MMF (*N* = 61) and SLS I-PBO (*N* = 61) participants	I: FVC (%); II: DLCO (%), PR-D	MMF in comparison with PBO was associated with an improved course of FVC (%) (*p* < 0.0001), DLCO% (*p* < 0.001), and PR-D (*p* = 0.0112) after FU period.
Nadashkevich et al. [15]	2006	60 SSc°	N.A.	18 Mo	CYC vs. AZA	Oral CYC [30 py] vs. oral AZA [30 pt]. PRD for the first 6 months.	I: FVC (%); DLCO (%), Chest X ray	FVC and DLCO did not change after treatment in the CYC-group, but statistically significantly worsened in the AZA-group.
Daoussis et al. [16]	2010	14 SSc-ILD	N.A.	12 Mo	RTX	RTX [8 pt] vs. PBO [6 pt]	I: FVC (%), FEV1 (%), DLCO (%); II: HRCT score	There was a significant increase of FVC and DLCO in the RTX group compared with baseline (*p* = 0.0018 and *p* = 0.017). HRCT scores were identical at baseline and at 24 weeks in all patients in the RTX group, while in the control group, there was a modest nonsignificant increase.
RECOVER trial [17]	2013	22 SSc	Phase II/III	12 Mo	RTX	RTX vs. PBO	II: Pulmonary functional tests	N.A.
ASTIS trial [18]	2014	156 SSc (n° SSc-ILD n.a.)	Phase II	24 Mo	HSCT vs. CYC	HSCT [79 pt] vs. CYC [77 pt]	I: event-free survival. II: FVC (%), TLC (%); RV (%); DLCO (%)	HSCT therapy resulted in significant improvement in the FVC and total lung capacity (TLC) at a two-year follow-up
ASSIST trial [19]	2011	19 SSc-ILD	Phase II	12 Mo	HSCT vs. CYC	HSCT [10 pt] vs. CYC [9 pt].	FVC (%); DLCO (%); HRCT score	HSCT in comparison to CYC was more effective in improving FVC and decreasing diseased-lung volume. No effects on DLCO (%) was observed.
SCOT trial [20]	2018	73 Ssc-ILD	Phase II/III	54 Mo	HSCT vs. CYC	HSCT [36 pt] vs. CYC [37 pt]	I: Global rank composite score including FVC (%)	HSCT achieved long-term benefits in patients with scleroderma, including improved event-free and overall survival Data regarding pulmonary function were not available.
SENSCIS trial [21]	2019	576 Ssc-ILD	Phase III	12 Mo	Nintedanib	Nintedanib [288 pt] vs. PBO [288 pt]	I:FVC (mL) ^; II: FVC (%) ^, FVC (mL) ^$^, DLCO (%)$ PR-D	Nintedanib significantly reduced the annual rate of decline in FVC at the end pf FU (*p* = 0.04), even if the relative reduction in FVC decline was similar in the two groups. Additionally, other pulmonary secondary outcomes were not reached.
Khanna et al. [22]	2011	20 SSc-ILD	Phase I/IIa	12 Mo	Imatinib	Imatinib [20 pt]	I: FVC (%), DLCO (%), HRCT, PR-D	Imatinib led to trends toward improvement of 1.74% in the estimated FVC %, TLC % and in the DLCO % predicted over a 1-year period (*p* not significant). PR-D improved statistically, but the improvement was not clinically meaningful.
Fraticelli et al. [23]	2014	30 SSc-ILD	Phase II	6 Mo *	Imatinib	Imatinib [30 pt] for 6 months	I: FVC (%), DLCO (%), HRCT, PR-D	Three patients died and one pt was lost to follow-up. Four pt had a good response, seven worsened and 15 had a stabilized lung disease. Overall, 19 pt had an improved or stabilized lung disease. After a 6-month follow-up, 12 (54.5%) of the 22 pt showed an improved or stabilized lung disease.
Martyanov et al. [24]	2017	31 SSc-ILD	Phase IIa	18 Mo *	Dasatinib	Dasatinib for 6 months	II: PFT, PR-D, HRCT, Serum KL-6, SP-D, APRIL and adiponectin.	No significant changes in clinical assessments or serum biomarkers were seen at the end of FU. By quantitative HRCT, 65% of patients showed no progression of FIB, 39% showed no progression of total ILD. Improvers showed stability in FVC and DLCO, while both measures showed a decline in non-improvers (*p* = 0.1289 and *p* = 0.0195, respectively).
LOTUSS trial [25]	2016	63 SSc-ILD	Phase II	5 We *	PRF	2-week titration [32 pt] vs. 4-week titration [31pt] from 801 mg/d to 2403 mg/d, for 16 weeks.	EE: FVC (%), DLCO (%)	FVC (%) and DLCO (%) remained largely unchanged at the end of FU.
SLS III [26]	2017	*Recruiting* (estimated 150 SSc-ILD)	Phase II	18 Mo	MMF, PRF	MMF +PBO vs. MMF + PRF	I: FVC (%); II: DLCO (%), PR-D, HRCT	N.A.
FaSScinate trial [27]	2016	87 SSc	Phase II	48 We	TCZ	TCZ [43 pt] vs. PBO [44 pt]	EE: FVC (mL and %) ^$^, DLCO (%) ^$^	FVC showed a not significant decrease in TCZ group than PBO group at the end of FU and fewer pt in the TCZ group than in the PBO group had worsening of percent predicted FVC (*p* = 0·037). The change from baseline in DLCO (%) did not differ significantly between PBO and TCZ.
FocuSSced trial [28]	2018	212 SSc	Phase III	48 We	TCZ	TCZ vs. PBO	II: FVC (%) ^$^	The cumulative distribution of change from baseline to week 48 in FVC (%) favored TCZ over PBO (*p* = 0.0015). The difference in mean change from baseline in FVC at week 48 was in favor of TCZ. Preservation of lung function with TCZ was shown by change from baseline in FVC over time.
Dermatomyositis and Polymyositis								
Allenbach et al. [29]	2015	12 ASSD	Phase II	12 Mo	RTX	RTX	II: Improvement of ILD (increase of 10% in FVC or 15% of DLCO %).	Improvement of FVC was observed in four patients, stabilization in 5 five and worsening in one. Only 1 pt with increased FVC (%) also showed an improvement of DLCO (%). In addition, one patient had an improvement of DLCO without significant change for FVC (data not shown). Finally, five patients had improving ILD measured by PFT.
ATtackMy-ILD [30]	2017	Recruiting (estimated 20 ASSD-ILD)	Phase II	6 Mo	ABA	ABA vs. PBO	I: FVC (%) ^$^; II: time to progression free survival, PR-D, time to improvement in FVC% (≥10 points)	N.A.
Connective tissue diseases								
RECITAL [31]	2013	Recruiting (estimated 116 CTD-ILD)	Phase II/III	48 We	RTX, CYC	RTX *vs.* CYC	I: FVC (mL); II: DLCO ^$^	N.A.
INBUILD trial [32]	2019	663 pt with progressive fibrosing ILD other than IPF (including CTDs)	Phase III	52 We	Nintedanib	Nintedanib vs. PBO	I: FVC ^; II: absolute change from baseline in the total score on the King’s Brief Interstitial Lung Disease (K-BILD) questionnaire; time until the first acute exacerbation of ILD or death;	In patients with progressive fibrosing interstitial lung diseases, the annual rate of decline in the FVC was significantly lower among patients who received nintedanib than among those who received placebo. Diarrhea was a common adverse event.

SLS: Scleroderma Lung Study; CYC: Cyclophosphamide; pt: patients; Mo: Month; FVC: Fored Vital Capacity; TLC: Total Lung Capacity; PR-D: patient-reported dyspnea; DLCO: diffusing capacity of the lung for carbon monoxide; N.A.: not available; I primary outcome; II secondary outcome; ^§^ Data extrapolated from a previous trial; * after the end of treatment; ° at the baseline 1% in each treatment group presented X-ray evidence of bibasilar pulmonary fibrosis. FVC and DLCO were evaluated in the overall patient group; AEs: adverse events; FU: follow-up; GGOs: ground-lass opacities; FIB: fibrosis; HCs: honeycomb cysts; PDN prednisone; Iv: intravenous; $ change from baseline; ^ annual rate decline; KL 6: Krebs von den Lungen-6; SP-D: surfactant protein D; APRIL: B cell proliferation-inducing ligand; We: weeks; EE: Exploratory endpoints; TCZ: tocilizumab; PRF: pirfenidone; PBO: placebo; ASSD: Anti-synthetase syndrome; ABA: Abatacept; CTD: connective tissue disease.

**Table 2:** Treatment of patients with CTD related ILD. Summary of the literature according to the study design.

	Systemic Sclerosis	Idiopathic Inflammatory Myopathy	Primary Sjogren Syndrome	Systemic Lupus Erythematosus
Glucocorticoids	Prospective [171] Nested case control [38] Retrospective [34]	Prospective [89] Retrospective [98,100,101,112]		
Cyclophosphamide	Randomized clinical trial [9,10,11,12,13,15,40] Prospective [171] Retrospective [34,41,50,52]	Prospective [89] Retrospective [79,88,91,115]		
Mycophenolate Mofetil	Randomized clinical trial [13,14] Prospective [45] Retrospective [34,43,44,46,47,48,51]	Retrospective [79,92,93]		
Azathioprine	Randomized clinical trial [12,15] Retrospective [34,50]	Prospective [89] Retrospective [79]		
Rituximab	Randomized clinical trial [16,17] Prospective [57,59] Nested case control [54] Retrospective [53,55,56,58]	Randomized clinical trial [29] Retrospective [119,120,121,122,123,125] Case report [109,115]	Prospective [143] Retrospective [145] Case report [136]	
Hematopoietic stem cells transplantation	Randomized clinical trial [18,19,20]			
Tyrosine kinase inhibitors	Randomized clinical trial [21,22,23,24,72]			
Pirfenidone	Randomized clinical trial [25,26] Prospective [73] Retrospective [74]			Case report [150]
Calcineurin inhibitors		Prospective [106] Retrospective [81,97,98,99,100,102,107,108,109,110,112,114,115,117]		
Lung transplantation	Retrospective [61,63,64,65,66,67,68,69,70]	Retrospective [133,134,135] Case report [131,132]		
Plasma exchange		Case report [129,130]		
Intravenous Immunoglobulins		Retrospective [126,128] Case report [127]		
IL-6 inhibitors	Randomized clinical trial [27,28]		Case report [146]	
Methotrexate		Prospective [89]		

IL: interleukin.
